# Transforming Poultry Farming: A Pyramid Vision Transformer Approach for Accurate Chicken Counting in Smart Farm Environments

**DOI:** 10.3390/s24102977

**Published:** 2024-05-08

**Authors:** Ridip Khanal, Yoochan Choi, Joonwhoan Lee

**Affiliations:** 1 Division of Computer Science and Engineering, Jeonbuk National University, Jeonju 54896, Republic of Korea; ridipk@gmail.com (R.K.); choihascold@gmail.com (Y.C.); 2 Department of Computer Science and Applications, Tribhuvan University, Mechi Multiple Campus, Bhadrapur 57200, Nepal

**Keywords:** smart farm environment, poultry farming, chicken counting, pyramid vision transformer, augmentation strategy, challenges in counting

## Abstract

Smart farm environments, equipped with cutting-edge technology, require proficient techniques for managing poultry. This research investigates automated chicken counting, an essential part of optimizing livestock conditions. By integrating artificial intelligence and computer vision, it introduces a transformer-based chicken-counting model to overcome challenges to precise counting, such as lighting changes, occlusions, cluttered backgrounds, continual chicken growth, and camera distortions. The model includes a pyramid vision transformer backbone and a multi-scale regression head to predict precise density maps of the crowded chicken enclosure. The customized loss function incorporates curriculum loss, allowing the model to learn progressively, and adapts to diverse challenges posed by varying densities, scales, and appearances. The proposed annotated dataset includes data on various lighting conditions, chicken sizes, densities, and placements. Augmentation strategies enhanced the dataset with brightness, contrast, shadow, blur, occlusion, cropping, and scaling variations. Evaluating the model on the proposed dataset indicated its robustness, with a validation mean absolute error of 27.8, a root mean squared error of 40.9, and a test average accuracy of 96.9%. A comparison with the few-shot object counting model SAFECount demonstrated the model’s superior accuracy and resilience. The transformer-based approach was 7.7% more accurate than SAFECount. It demonstrated robustness in response to different challenges that may affect counting and offered a comprehensive and effective solution for automated chicken counting in smart farm environments.

## 1. Introduction

Chicken counting in smart farm environments uses artificial intelligence and computer vision to count chickens automatically. Smart farm environments are agricultural systems that use advanced technologies, such as sensors, actuators, cameras, and cloud computing to monitor and control various aspects of farming operations, including growth, health, and resource management [1]. This research attempts to optimize chicken growth on poultry farms. It specifically focuses on critical aspects of chicken counting because this has direct and indirect implications on identifying the ideal chicken density, discerning patterns in chicken density, determining the precise quantities of food and water required, establishing optimal environmental conditions for the birds’ growth, and understanding the different stages of their development. By examining chicken count, it will be possible to gain a deeper understanding of poultry farming, paving the way for more sustainable and effective production methods. Chicken counting also helps to maintain the primary parameters in poultry farming, including temperature, humidity, ammonia concentration, air quality, light intensity, and litter moisture [2]. These factors are directly linked to chicken health and, thus, to productive chicken farming.

This research studies the interface of technology and agriculture and attempts to identify best practices for chicken farming. Through chicken counting and analysis, it aims to enhance agricultural conditions, improve animal welfare, and develop sustainable farming techniques. By embracing the potential of smart farm chicken counting, it heralds a new era of efficient, ethical, and sustainable poultry farming, providing a superior farm-to-table experience for consumers. Smart farm chicken counting is a crucial component of efficient livestock management and offers farmers and consumers several benefits. It supports improvements in productivity and profitability on chicken farms. It can help farmers to optimize the number of chickens in each coop or barn, which can affect the feed conversion ratio and the chickens’ growth and mortality rates [3]. Knowing the exact number of chickens in each flock can also be helpful for farmers to plan for the market’s supply and demand, thus avoiding over- or underproduction. In addition, automating chicken counting can reduce the stress and discomfort caused by manual counting, which disturbs the chickens and exposes them to human contact and potential pathogens. By continuously monitoring the number of chickens on the farm, farmers can detect any abnormal changes in the population, such as deaths, diseases, or escapes, and take timely actions to prevent further losses or outbreaks [4]. Furthermore, chicken counting is essential for promoting environmental and sustainable farming practices by reducing waste of feed, water, energy, and land. By integrating chicken counting with other smart farm technologies, such as RFID tags, GPS trackers, or blockchain platforms, farmers can also provide transparent and reliable information about their products’ origin, quality, and safety to consumers and regulators [5]. This can enhance the trackability benefits of food for both consumers and regulators.

**Figure 1:**
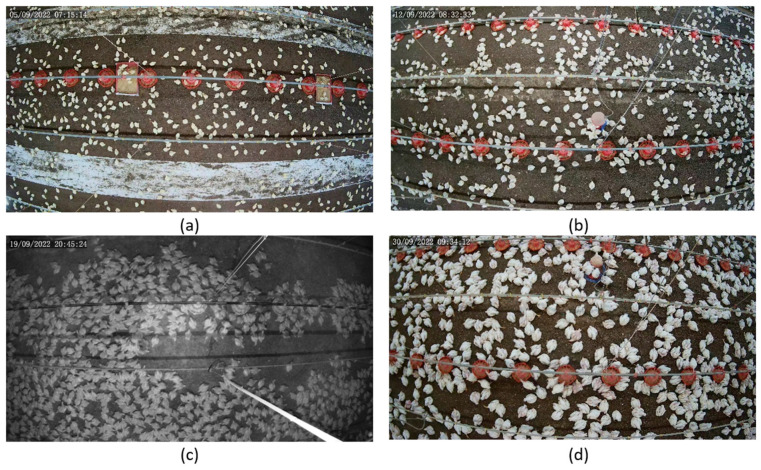
Examples of challenges in chicken counting: occluding and cluttering feeding plates and pipelines, camera distortion (particularly in the image corners), varied chicken sizes (in all images), white paint on the floor (**a**), smaller sized chickens (**b**), poor illumination (**c**), and high chicken density (**d**).

Counting chickens in a smart farm environment presents numerous challenges that require different techniques to overcome. As shown in Figure 1, some of the primary challenges are illumination changes, occlusions, cluttered background, continuous chicken growth, and camera distortions. The lighting conditions on a smart farm can vary according to the time of day, weather, season, or artificial sources. This can affect the visibility and contrast of the chickens in the images captured by the cameras and make it difficult to distinguish the birds from the background or each other. Shadows, glare, and reflections can cause some chickens to appear darker or brighter than others or to blend in with the surroundings [6]. Similarly, the chickens can be partially or fully occluded by various objects or structures, such as feeding plates, pipelines, or other chickens. This can reduce the visibility and completeness of the chickens in the images, making them hard to detect and count accurately; for example, some chickens can be hidden behind other chickens or objects or only show a part of their body, such as their head or tail [7]. The background can be cluttered with various elements and the background color can create visual noise, making it challenging for automated systems to accurately detect chickens. Distracting elements interfere with the identification process, and vibrant or patterned background colors can obscure chicken outlines, impacting the efficiency of the counting system. Another challenge is that chickens constantly grow and change their size, shape, and appearance. This can affect the consistency and reliability of the features used to identify and count them in the images. For example, some features, such as color, texture, or pattern, may change as the chickens grow feathers or molt. Some features, such as size, shape, or posture, may vary depending on the age, breed, or health. Finally, camera lenses can introduce distortions like radial or tangential perspectives to images, especially towards the edges of the frame [8]. This can warp the shape of the chicken or cause the image to tilt or skew, making it challenging to locate and count chickens accurately.

This study proposes and evaluates a novel method for counting chickens in smart farm environments, using a deep learning (DL) approach based on transformer architecture. It believes that transformers are effective for chicken counting due to their ability to capture complex relationships, handle varying object sizes, and adapt to changing environmental conditions. The proposed method aims to address the challenges associated with chicken counting and provide accurate and reliable results for farm management. The proposed method consists of two main components: a pyramid vision transformer (PVT) backbone and a multi-scale regression head, along with a customized loss function incorporating curriculum loss. The PVT backbone captures the global crowd information from the input images, using a self-attention mechanism and patch embedding. The multi-scale regression head predicts density maps from the features extracted by the transformer backbone, using multi-scale dilated convolution (MDC) and pyramid feature aggregation (PFA). Incorporating curriculum loss into the model fosters a learning process that progressively adjusts to challenges presented by diverse chicken crowd scenarios, such as variations in densities, scales, and appearances. The proposed method can handle various conditions that may affect the visibility and completeness of the chickens in the images, such as illumination changes, occlusion, and continuous chicken growth. 

The method was evaluated on a newly created dataset containing annotated images from a smart chicken farm. The dataset contains images of chickens of varying sizes, in multiple positions and differing densities, under changeable lighting conditions. The method demonstrated robustness in response to different challenges affecting chicken counting in smart farm environments. The main contributions of this study are as follows:

- It proposes a novel and effective method for counting chickens in smart farm environments, using a DL approach based on transformer architecture and customized loss function incorporating curriculum loss.
- It addresses the challenges associated with smart chicken counting, such as illumination changes, occlusion, cluttered background, continuous growth, and camera distortion.
- It evaluates the proposed method on a newly created dataset and shows that it can achieve high performance efficiency and robustness for chicken counting in smart farm environments.

## 2. Related Works

Conventionally, chicken counting is performed manually, a time-consuming effort with imprecise results [9]. Traditionally, animal counting was undertaken using techniques like transect sampling, point counts, line transects, nest counts, dung or scat surveys, and acoustic surveys. In addition to these, there were techniques such as the mark-recapture method, remote sensing marking technology, and environmental DNA analysis. These manual processes are extremely expensive, time-consuming, tedious, and monotonous.

The use of artificial intelligence, such as autonomous equipment with machine learning (ML) algorithms [10] and/or DL tools and techniques [11], can improve the counting process. These algorithms and tools can be used to train a model for real-world contexts. Automatic counting uses two main processes: first, extraction of features from the target objects, like texture, shape, color, and size; second, objects are then detected based on these features, followed by the counting operation. Usually, ML algorithms like k-means [12], random forest [13,14], and support vector machine [15,16] are used. The second approach is to use the ML algorithms on the images based on DL concepts [11]. DL is a discipline within ML. It uses hierarchically structured multiple layers to extract useful representations. DL-based counting is implemented using different methods, including direct counting, detection, segmentation-based counting, and density-estimation-based counting.

Direct counting directly regresses the number of objects without relying on the precise locations of the counting. It is usually faster and simpler than other methods but suffers from low accuracy and robustness when the objects are small, occluded, or overlapping [17]. It can be accomplished using a classifier that incorporates domain knowledge to determine the number of classes for the classification. Häni et al. [18] employed a Resnet50 classifier [19] to count apples within image patches encompassing apple clusters. A more robust approach for direct counting involves using a deep regressor, which is a deep convolutional neural network equipped with a regressor head. Dobrescu et al. [20] employed a direct regressor that was trained on a restricted number of images to count leaves in a leaf-counting challenge. More complex pipelines with a direct regressor and different loss functions have also been implemented. Bhattarai and Karkee [21] proposed a regression-based network (CountNet) to count fruits and flowers in outdoor environments, using only image-level count annotation.

Object-detector-based counters use an object detector to detect and then count the target objects in an image. They operate by identifying the location of objects in the image and then classifying them into specific target classes [22]. This method looks natural, but the annotation for object-detection-based counting is time-consuming. Segmentation-based counters use an object segmentation module, which not only detects objects but also segments them before counting. It works by identifying the location of objects in the image, categorizing them into predefined target classes and segmenting the specific area of each identified object [23]. Hong et al. [24] used object detectors to detect and count relatively small insects (*Matsucoccus thunbergianae*) caught in insect traps. Ni et al. [25] trained a Mask-RCNN [26] model for segmenting blueberries within clusters to forecast yield. They employed a linear regression model to assess the model’s accuracy in detecting blueberries by comparing manual counts with the predicted number in each cluster. 

Counters based on density estimation employ a regression approach, wherein the network endeavors to estimate the count of the target object. This is achieved by predicting a heat map representation of the image, followed by regressing the number of objects from the generated heat map [27]. During the training process, the neural network is given a set of images along with annotated points that indicate the center of the target object in each image. The network is guided using these points to predict the heat map. The annotation burden is highly reduced, as the annotator simply needs to annotate a single point at the center of the target object. Tian et al. [28] used a convolutional neural network (CNN) model to acquire mapping between image features and density maps. This integrated approach allowed them to estimate count the number of pigs in an image captured on a pig farm. Gomez et al. [29] found that counting by regression becomes steadily more accurate than counting by detection when the object density increases. Other examples applying density estimation networks include Hobbs et al.’s [30] density estimation for automated pineapple flower counting from aerial imagery, Rahnemoonfar et al.’s [31] DisCountNet, and Xiong et al.’s [32] TasselNetv2, which involved using context-augmented local regression networks for the counting of wheat spikes.

Transformers can be used for counting, leveraging their parallel processing capabilities, attention mechanisms, and ability to capture long-range dependencies to accurately estimate crowd density in diverse and complex scenes. Sun et al. [33] used a transformer encoder–decoder to extract global context features from image patches. They introduced a token attention module to enhance the target object’s features with channel-wise attention and a regression token module to predict the total count. The framework outperformed the state-of-the-art methods on various benchmarks, including NWPU-Crowd [34], which is a challenging dataset for crowd counting. Yu et al. [35] used an interactive network based on a transformer for multimodal crowd counting, which can fuse the image and depth information of crowd scenes. The network consists of a sliding convolution encoding module, a main interactive network module, a token attention module, and a multilevel feature fusion module. The network captures the global and local features of crowd scenes and enhances these features with the token attention mechanism. The network achieved impressive results on the FSC-147 dataset [36], which is a large-scale dataset for multimodal crowd counting.

Curriculum learning, as proposed by Bengio et al. [37], is a training strategy in machine learning that draws inspiration from the human learning process. It gradually exposes the model to knowledge, starting with simple concepts and progressing towards more complex ones. Lui et al. [38] employed curriculum learning for crowd counting by designing a curriculum to feed the training images. Lyu and Tsang [39] proposed curriculum loss that can adaptively select samples for model training. Wang and Breckon [40] used curriculum loss for crowd counting in order to be aware of the pixel-wise difficulty level when computing the density map loss. 

Efforts have been made to automate chicken counting on farms. Cao et al. [7] used a localization-based counting algorithm known as LC-DenseFCN [41] to count chickens. This DL model used the efficient ResNet-50 and the point supervision algorithm to count chickens through a surveillance camera. The model employs a convolutional network to generate a density map and a point head module to refine the density map using an attention mechanism. They also used location-based counting loss, which supports the model by providing a semantic label for each pixel, separating the areas with multiple objects and removing the areas without objects. Abuaiadah et al. [42] applied a localized fully convolutional network algorithm to images of chickens for automated counting and grouping. Zhu et al. [43] used the YOLOv5 [44] model to automate chicken counting. They set the intersection over the union threshold by analyzing the width and height of the ground truth boxes of the training images. They also used mosaic, horizontal flipping combined with lightness changing, and test time augmentation to diversify the training data. Sun et al. [45] presented a chicken counting method utilizing YOLOv5 [44] and camera footage to monitor mixed Silkie and Xianghuang chickens in a large-scale environment.

Based on the methods described above, this study utilizes a transformer-based model because of its demonstrated benefits in managing complicated visual data. By harnessing transformers’ natural advantages, this study aimed to navigate the challenges associated with chicken counting to obtain reliable and precise results. In addition, to enhance the adaptability and precision of the chicken-counting model, the curriculum loss is incorporated, allowing the model to progressively learn from simpler to more complex examples, effectively addressing varying chicken densities, scales, and appearances.

## 3. Method

### 3.1. Chicken Counting Dataset

**Figure 2:**
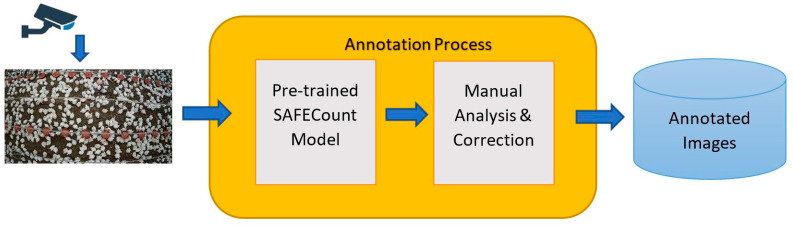
Image capturing and annotation pipeline.

A supervised chicken-counting dataset was created using real-time CCTV footage from a chicken farm and semi-automated annotation was conducted with SAFECount [46]. The focus was on creating diversity by capturing the images’ varying lighting conditions, as well as different chicken sizes, densities, and positions. For annotation, as shown in Figure 2, the unlabeled image was passed into the pre-trained SAFECount model to annotate the chickens automatically. The automatically annotated results were analyzed manually to correct incorrect annotations. Each annotation contained boundary box chicken coordinates (*x_min_*, *y_min_*, *x_max_*, *y_max_*) and was used to calculate the center coordinates (*x*, *y*). Center coordinates (*x*, *y*) were calculated as follows:

(1) $$x=\frac{x_{min}+x_{max}}{2}$$

(2) $$y=\frac{y_{min}+y_{max}}{2}$$

This dataset, comprising 56 images and their ground truths, was subsequently partitioned into training, validation, and testing sets. Due to the complexity and time-consuming nature of the annotation process, the dataset size was limited. To overcome this challenge and improve the model’s robustness, augmentation strategies were applied to the training data to increase their diversity and quantity. To address the challenges of poor illumination, partial occlusion, cluttered backgrounds, continuous chicken growth, and camera distortion, as well as to increase the volume of training data, two sets of data based on different augmentation strategies were constructed. In the first strategy (Strategy A), specific locations in the image, such as feeding plates and corners, were deliberately rotated to generate additional images. In the second strategy (Strategy B), in addition to rotations, augmentations like variations in brightness and contrast, shadow, blur, partial occlusion, random cropping, and scaling were added. Strategy A contained 2290 training data, while Strategy B contained 6059. These two sets of data were constructed to compare how successfully the aforementioned challenges were addressed. (See Table 1).

During augmentation, the rotation of the images at different angles was used to simulate different orientations of chickens. The brightness and contrast of the images were altered to account for different lighting conditions and help the model to become more robust to illumination changes. Random shadows were added to simulate shadows caused by obstructions or various lighting angles. Random blur was added to simulate the effects of background clutter and imperfect imaging conditions. Artificial occlusions were introduced to help the model learn how to handle partial obstructions. Random cropping was applied to simulate chickens occupying different parts of the frame so that the model could focus on specific areas of the image. Scaling (resizing the images to different scales) was used to simulate the chickens being at various distances from the camera.

### 3.2. Transformer-Based Chicken Counting Architecture

The chicken-counting model, inspired by CCTrans [47], builds upon its architecture with a transformer backbone to capture the global context of chicken images. Furthermore, the model was enhanced through the incorporation of a customized loss function, including the addition of curriculum loss [39,40], to optimize performance and adapt to varying chicken densities, scales, and appearance. In comparison to CNN [48], which has limited receptive fields and relies on local features, transformers can model long-range dependencies and global information among the pixels.

**Figure 3:**
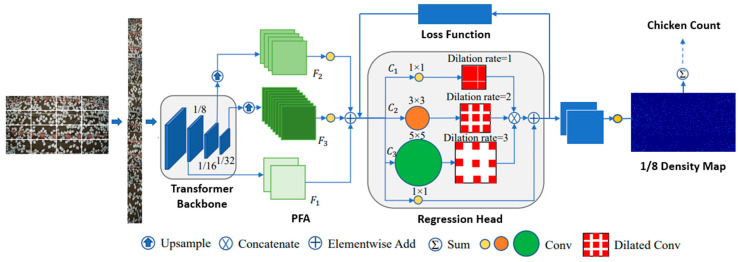
The pipeline of the transformer-based chicken-counting model.

As shown in Figure 3, the architecture consists of four main components: a pyramid vision transformer (PVT), a pyramid feature aggregation (PFA) module, an efficient regression head, and a set of tailored loss functions. The PVT generates multi-scale features from the input image and is composed of four stages, each with a different number of transformer blocks and spatial resolution. The transformer blocks use self-attention and feed-forward networks to process the image patches as tokens. The PVT can handle different levels of chicken density and scale by producing features with different granularities. The PFA module fuses the low- and high-level features from different PVT stages and consists of two sub-modules: a feature-fusion sub-module and a feature-refinement sub-module. The feature-fusion sub-module uses element-wise addition to combine the features from adjacent PVT stages, while the feature-refinement sub-module uses convolutional layers to enhance the fused features. The PFA module improves the feature representation and preserves the spatial information of the chicken scenes. The efficient regression head is used to predict the density maps of the chicken scenes, which can reflect the number and location of the chickens. The regression head uses multi-scale dilated convolution (MDC) to process the features from the PFA module. The MDC can enlarge the receptive field and maintain the spatial resolution without increasing the computational cost. The regression head outputs a density map for each scale level, which is then summarized to obtain the final density map. 

The counting model uses a combination of counting loss, optimal transport (*OT*) loss, total variation (*TV*) loss, and curriculum (*CC*) loss. Counting loss quantifies the discrepancy between the predicted and ground truth crowd counts, focusing on accurately estimating the number of chickens in the image. *OT* loss measures the similarity between the predicted and ground truth density maps by computing the minimum transportation cost required to transform one distribution into another. It helps in aligning the spatial distribution of predicted and ground truth density maps. *TV* loss penalizes spatial variations in the predicted density map, encouraging smoothness and coherence in the density predictions. It aids in minimizing abrupt changes or inconsistencies in the density distribution. The *CC* loss is adapted to enhance the model’s progressive learning, addressing challenges posed by varying densities, scales, and appearances during chicken counting. It dynamically adjusts the learning objectives during training to address these associated challenges. By integrating these loss components, the model learns to effectively count chickens while maintaining spatial coherence and consistency in density predictions, leading to more accurate and reliable results. For a predicted density map *D* and its ground truth *D’*, the loss function is defined as follows:

(3) $$L=L_{1}\;(P,\;G)+\lambda_{1}L_{OT}+\lambda_{2}L_{TV}\;(D,D’)+\lambda_{3}L_{CL}$$

where *P* and *G* denote the chicken count of *D* and *D’*, respectively. *λ*_1_, *λ*_2_, and λ_3_ are the loss coefficients.

This architecture is used for the counting model because it boasts several features and advantages. Firstly, it employs transformers, which are particularly good at capturing global information and long-range interdependence in crowd images. This capacity is essential for precisely counting chickens dispersed over a large region. Second, the PVT produces multi-scale features that adapt to different crowd densities and scales. This adaptability is crucial since chicken densities and sizes might vary on a farm. Third, the PFA module combines high- and low-level features. This fusion guarantees precise chicken counting even in complex settings by preserving spatial information and improving feature representation. Fourth, the use of MDC and an efficient regression head allows the network to predict density maps with expanded receptive fields while maintaining spatial resolution. This results in precise chicken localization and counting. Finally, the customization of the loss function, by incorporating curriculum loss, facilitates progressive learning, enabling adaptation to diverse complexities such as varying chicken densities, scales, and appearances. This approach enhances the model’s generalization, mitigates annotation challenges in weakly-supervised settings, and contributes to improved convergence.

In summary, the transformer-based method combined with the customized loss function is notable for its ability to handle diverse chicken densities, scales, and spatial complexities. By leveraging its global-context modeling, multi-scale features, efficient fusion techniques, and versatile training methods, it is well-suited to handle the complexities of automated chicken counting in varying farm environments, making it an excellent choice for our model.

### 3.3. Evaluation Metrics

The model was evaluated using the mean absolute error (*MAE*), root mean squared error (*RMSE*), and average accuracy (*AA*) metrics. The *MAE* measures the average difference between the predicted and ground truth chicken counts. The *RMSE* measures the square root of the average of the squared difference between the predicted and ground truth chicken counts. Lower *MAE* and *RMSE* values indicate better model performance. The *AA* evaluates the accuracy of the model’s count predictions compared to the ground truth counts. A higher *AA* value indicates better model performance. These metrics are formulated as follows:

(4) $$MAE=\frac{1}{N}\sum_{i=1}^{N}|{MP}_{i}-{GT}_{i}|$$

(5) $$RMSE=\sqrt{\frac{1}{N}\sum_{i=1}^{N}({MP}_{i}-{GT}_{i}{)}^{2}}$$

(6) $$AA=\frac{1}{N}\sum_{i=1}^{N}\left(1-\frac{|{GT}_{i}-{MP}_{i}|}{{GT}_{i}}\right)$$

where *N* is the number of test images, *MP_i_* is the model prediction count, and *GT_i_* is the ground truth count for the *i*-th observation.

## 4. Experimental Results

### 4.1. Results

The chicken-counting model trained on the original dataset (non-augmented) achieved 47.4 MAE and 75.8 RMSE on the validation set, 40.7 MAE and 63.2 RMSE on the test set, and an AA of 0.9429 on the test set. On the data from the dataset augmented using strategy A, the model achieved 34.1 MAE and 49.2 RMSE on the validation set, 32.6 MAE and 54.4 RMSE on the test set, and an AA of 0.9546 on the test set. This augmentation strategy increased the AA by 1.2%. Similarly, on the data from the dataset augmented using strategy B, the model achieved 27.8 MAE and 40.9 RMSE on the validation set, 22.0 MAE and 32.3 RMSE on the test set, and an AA of 0.9696 on the test set. This augmentation strategy increased the AA by 2.7% as compared to non-augmentation, and by 1.5% as compared to strategy A. (See Table 2).

To assess the effectiveness of the customized loss function incorporating curriculum loss, the model was also trained on the dataset (augmented using strategy B) without using the curriculum loss. The results from this training achieved 40.3 MAE and 58.9 RMSE on the validation set, 31.9 MAE and 47.8 RMSE on the test set, and an AA of 0.9555 on the test set. In contrast, the chicken-counting model incorporating curriculum loss achieved better results in terms of MAE and RMSE, and improved AA by 1.4%. (See Table 3).

**Figure 4:**
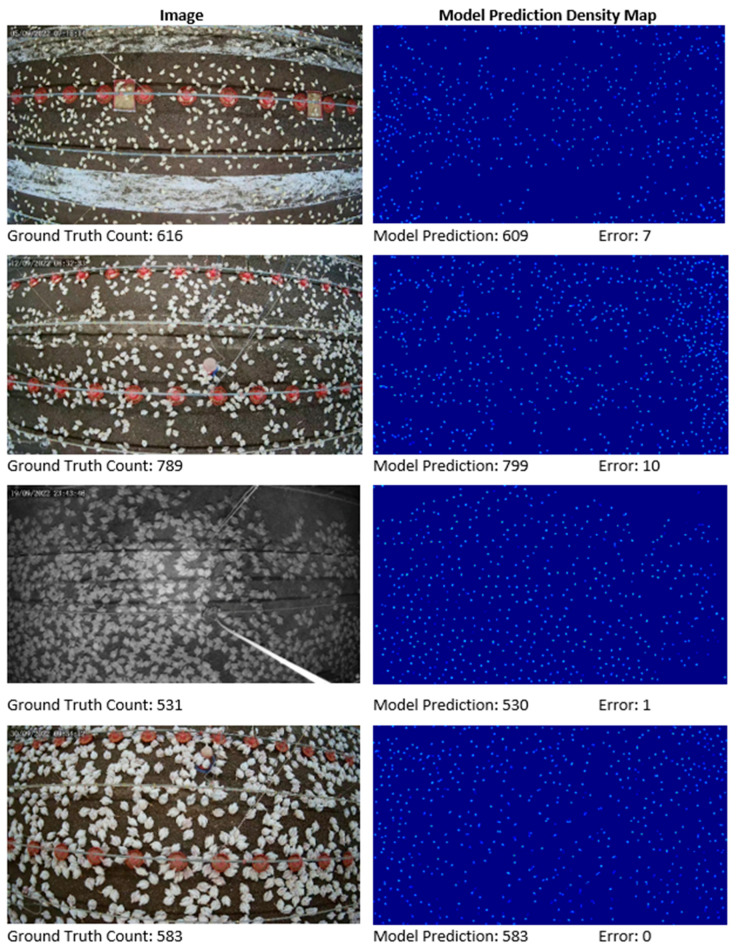
Visualization results of the chicken-counting model on a newly created chicken dataset. Images with varying chicken sizes, densities, and lighting conditions are shown with their ground truth count, model prediction, and error, which is the absolute difference between ground truth and model prediction.

The chicken-counting model was trained using a crop size of 256, an AdamW optimizer [49] with a batch size of eight, an initial learning rate of 1 × 10^−5^, and an L2 regularization of 0.0001 for 1500 epochs. All experiments were conducted using PyTorch version 1.10.0 installed with CUDA 11.3 support, employing NVIDIA GeForce RTX 3090 GPU from NVIDIA Corporation (Nvidia, Santa Clara, CA, USA). (See Figure 4).

### 4.2. SAFECount Comparison

SAFECount [46] is a few-shot object-counting model that uses a similarity-aware feature enhancement block for counting the number of exemplar objects occurring in the query images. It consists of a similarity comparison module and a feature enhancement module. The similarity comparison module computes the similarity map that indicates the probability of each pixel belonging to the target class, using a point-wise feature comparison of the support and query features and then normalizing it across exemplar and spatial dimensions to produce a reliable similarity map. The feature enhancement module is responsible for using the similarity map to enhance the query feature with the support features. This helps the model to detect distinguishable borders between densely packed objects.

Both SAFECount and transformer-based object counting are efficient and scalable, and have achieved accurate results on various benchmarks for object counting. However, transformer-based object counting is slightly superior in terms of accuracy and robustness, as it captures and transfers the global context and semantic features of the images across different domains using the transformer architecture. In addition, the use of curriculum loss optimizes performance and adapts to varying conditions. The use of SAFECount relies on similarity comparison and feature enhancement modules to improve the discrimination and edge recovery of objects, so it may not be able to handle complex scenes or diverse object categories as well as transformer-based models. On the other hand, the transformer-based model has a higher computational cost, as it uses more parameters and floating-point operations per second (FLOPs).

SAFECount was also trained on our dataset, attaining 64.5 MAE and 112.5 RMSE on the validation set, 59.4 MAE and 100.6 RMSE on the test set, and an AA of 0.8922 on the test data. Our transformer-based counting model achieved better results in terms of MAE and RMSE, improving AA by 7.7%. (See Table 4).

## 5. Discussion

**Figure 5:**
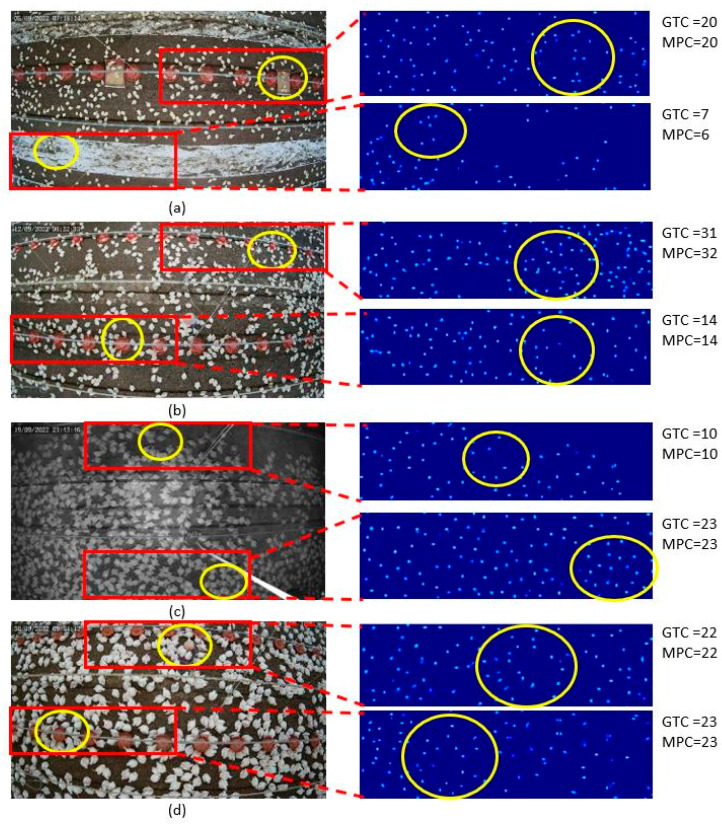
Qualitative results presenting the model addressing the challenges of chicken counting. GTC represents the ground truth count and MPC represents the model prediction count of the chickens within the yellow circle. Chickens were detected when their size varied and the camera was distorted (all images), when the background was cluttered by white paint on the floor and objects (feeding plates and pipelines) (**a**), with the presence of occlusions due to pipelines and feeding plates (**b**,**d**), and in low lighting conditions (**c**).

The results of the proposed chicken-counting model, as demonstrated with 27.8 MAE and 40.9 RMSE on the validation set and 22.0 MAE and 32.3 RMSE on the test set, underscore the precision of our approach (see Section 4.1 for detailed results). The model exhibited efficacy even under challenging conditions such as varying illumination, cluttered backgrounds, partial occlusions, continuous chicken growth, and camera distortion. Qualitative results, depicted in Figure 5, visually corroborate these findings.

The achieved accuracy of 0.9696 on the test data further substantiates the reliability of the model in real-world farming situations. It is important to note that accuracy values closer to unity (1.0) indicate superior performance, reflecting a higher percentage of correctly identified chicken instances compared to ground truth annotations. Augmentation strategy B, simulating challenging counting conditions, not only enhanced the model’s adaptability but also increased its accuracy by an additional 2.7% (see Section 4.1 for detailed results).

Addressing the primary challenges (see Figure 1 for detailed challenges) is crucial in automated chicken counting, as these factors significantly impact the visibility and the appearance of chickens in images. The proposed method demonstrated robustness in overcoming these challenges by leveraging its transformer backbone [33]. In contrast to traditional methods, which rely on local features from CNNs [48], the transformer captures long-range dependencies in the pixel relationships, allowing it to understand the global context of crowd scenes and discern the semantic relationships between chickens and their surroundings. This capability enables the model to identify occluded chickens and adapt to diverse lighting conditions. The transformer-based approach, through its adept handling of long-range dependencies, substantially enhances the accuracy and efficiency of automated chicken counting in various farm environments.

The customized loss function, employing curriculum loss, plays a vital role in training the model effectively. It serves as a valuable strategy to enhance learning and adaptability. It supports progressive learning, adaptation to diverse challenges, effective handling of varying densities, improved generalization, enhanced training stability, and fine-tuning of the model’s sensitivity. The results of incorporating this loss function underscore its significance as a valuable component in optimizing the performance of the model. 

The proposed method is noteworthy for its adaptability to diverse challenges in smart farm environments. The model addresses issues such as varying lighting conditions, occlusions, cluttered backgrounds, continuous chicken growth, and camera distortion. Leveraging the PVT and PFA modules [47], the customized loss function of the model excels in handling different chicken densities and sizes, making it well suited for the dynamic conditions of poultry farming. 

The comparison with SAFECount [46] reinforced the superiority of our transformer-based approach. Regarding object counting, both models are efficient, scalable, and accurate, but the transformer-based model exhibited higher accuracy and robustness. Its capacity to capture and transfer global context and semantic features across different domains, thanks to the transformer architecture, gives it a significant advantage. Despite the higher computational cost associated with more parameters and FLOPs, the transformer-based model outperformed SAFECount.

The transformer-based chicken-counting model introduced in this study holds significant promise for smart farm operations. By addressing the complexities of accurate chicken counting, the model contributes to more efficient livestock management, optimizing factors such as growth rate, feed conversion ratio, and mortality rate [3]. This helps farmers to respond to market supply and demand. Automated counting also minimizes the stress on chickens and the potential health risks associated with manual counting.

This research propels smart farm practices into a new era of precision and sustainability. Accurate chicken counting facilitates optimal resource utilization, promoting environmental consciousness and ethical farming. Implementing the potential of transformer-based models in smart farm environments promises a superior farm-to-table experience, ensuring both productivity and animal welfare in poultry farming.

## 6. Conclusions

In summary, the model offers a comprehensive and effective solution for automated chicken counting in smart farm environments. The proposed method, evaluated on a diverse dataset and validated through comparison with the SAFECount object counting model, demonstrated its efficacy in overcoming counting challenges unique to poultry farming. Through its adaptability, precision, and robustness, our model contributes significantly to the advancement of efficient, ethical, and sustainable practices in poultry farming, fostering a superior farm-to-table experience for consumers.

We obtained an impressive 27.8 MAE and 40.9 RMSE on the validation set, 22.0 MAE and 32.3 RMSE on the test set, and an AA of 0.9696 on test data, demonstrating the model’s robustness and accuracy in various farming scenarios. The augmentation strategies employed, particularly strategy B, enhanced the model’s adaptability to challenging conditions, resulting in a 2.7% increase in accuracy.

## Figures and Tables

**Table 1:** Details of three datasets used.

Dataset	Number of Training Images	Augmentation Strategy Used
Original	36	None
Strategy A	2290	Rotation around feeding plates and corners
**Strategy B**	**6059**	**Rotations, variations in brightness, contrast, shadow, blur, partial occlusion, random cropping, and scaling**

**Table 2:** The counting model’s performance on three datasets.

Dataset	Val Set		Test Set		AA
	MAE	RMSE	MAE	RMSE	
Original (non-augmented)	47.4	75.8	40.7	63.2	0.9429
Augmented using Strategy A	34.1	49.2	32.6	54.4	0.9546
**Augmented using Strategy B**	**27.8**	**40.9**	**22.0**	**32.3**	**0.9696**

**Table 3:** Performance comparison between before and after using curriculum loss.

Customized Loss Function	Val Set		Test Set		AA
	MAE	RMSE	MAE	RMSE	
Without using Curriculum Loss	40.3	58.9	31.9	47.8	0.9555
**Using Curriculum Loss (ours)**	**27.8**	**40.9**	**22.0**	**32.3**	**0.9696**

**Table 4:** Performance comparison between our chicken-counting model and SAFECount.

Method	Val Set		Test Set		AA
	MAE	RMSE	MAE	RMSE	
SAFECount	64.5	112.5	67.9	104.2	0.8922
**Chicken-Counting Model (ours)**	**27.8**	**40.9**	**22.0**	**32.3**	**0.9696**

## Data Availability

Data are contained within the article.
